# Year-end reflections of EVCNA-2021

**DOI:** 10.20517/evcna.2021.27

**Published:** 2021-12-31

**Authors:** Y. Peng Loh

**Affiliations:** American Biochemist and Molecular Biologist, Bethesda, MD 20817, USA.

*Extracellular Vesicles and Circulating Nucleic Acids* (*EVCNA*) was conceived in 2019, and the first issue was launched in December 2020, which included a report of presentations, abstracts, and contributed articles from the American Society for Extracellular Microvesicles 2020 meeting. In 2021, the international editorial board of the journal grew to 40 members from 15 countries with 5 Associate Editors. In addition to the Editorial Board comprising of eminent scientists covering many aspects of research in extracellular vesicles and circulating nucleic acids, we have an international junior editorial board of 40 young early career scientists, providing an opportunity for them to participate in editorial duties. In 2021, the journal published 4 quarterly issues with 16 articles, including original research, reviews, commentaries, and conference report. Ten of the articles have been cited 22 times in SCI journals in this one year. Manuscript submissions were thoroughly peer reviewed, and our rejection rate in 2021 was 29%. Our readership in 2021 was diverse spanning more than 15 countries worldwide with more than 28,814 views and 5542 downloads.

In 2021, the editorial team and OAE Publishing Inc., the publisher of *EVCNA*, have partnered with various Extracellular Vesicle and Liquid Biopsy Societies from different countries, including the German Society for Extracellular Vesicles, Italian Society for Extracellular Vesicles, USA, and China CACA TBM Society for Exosomes and Microvesicles. *EVCNA* has also sponsored awards to promote the careers of young investigators and honored a keynote speaker with *CSEMV* & *EVCNA* Best Lectureship Award at the Chinese Academic Conference on tumor markers and the 15th Young Scientists Forum on tumor markers. Prof. Hang Yin of Tsinghua University is the first recipient of *CSEMV* & *EVCNA* Best Lectureship Award.

*EVCNA* has posted short videos highlighting the work of many authors on the journal website and social media. Those have been well received from the feedback.

Looking forward to 2022, *EVCNA* has at least 6 Special Issues in progress that will be published in the coming year. They cover broad areas of EV research, including the following titles:

“Extracellular Vesicles and Neuroinflammation: Response to Infection, Injury and Aging, Plus Vehicles for Treatment” (Guest Editor: Lynn Pullman); “Exosomes: Diagnostics, Delivery and Therapeutics in Cancer” (Guest Editor: Long Zhang) and “Clinical Use of Circulating Nucleic Acids in Bodily Fluids: Noninvasive Prenatal Testing, Oncology, Transplant Monitoring and Beyond” (Guest Editors: Erik Sistermanns and Peiyong Jiang). It is still possible to contribute an article to any of our Special Issues listed on our website, just let the Guest Editor(s) know. We will also have a Special Issue on the Proceedings of the American Society for Intercellular Communications 2021, highlighting a report of the exciting science presented, abstracts, and contributed articles of work presented at the meeting. Finally, in the coming year, *EVCNA* will be organizing some virtual workshops and mini-symposiums on hot topics of interest to researchers in the EV and circulating nucleic acid field. In particular, we aim to promote translational research focusing on EVs and Nucleic acids as biomarkers and EVs in therapy. EVs and Liquid Biopsy continue to be an exciting field, and there is a lot we still don’t know. It is hoped that *EVCNA* will continue to be a platform of choice to share your scientific data, knowledge, and opinions with fellow researchers in this field. For now, this journal has waived APCs, providing a great opportunity for everyone to be able to publish their experimental data or scholarly reviews and commentaries freely.

In 2021, *EVCNA* has enjoyed great support from the scientific community working in this field, and we thank everyone for their contributions. We look ahead to greater success in 2022 and serving the researchers in the field of Extracellular Vesicles and Liquid Biopsy.

Warm Wishes to everyone for Good Health and Success for 2022.

## DECLARATIONS

### Copyright

© The Author(s) 2021.

